# Is PSMA PET/CT cost-effective for the primary staging in prostate cancer? First results for European countries and the USA based on the proPSMA trial

**DOI:** 10.1007/s00259-023-06332-y

**Published:** 2023-07-10

**Authors:** Adrien Holzgreve, Marcus Unterrainer, Jérémie Calais, Thaiza Adams, Daniela E. Oprea-Lager, Karolien Goffin, Egesta Lopci, Lena M. Unterrainer, Kristina K. M. Kramer, Nina-Sophie Schmidt-Hegemann, Jozefina Casuscelli, Christian G. Stief, Jens Ricke, Peter Bartenstein, Wolfgang G. Kunz, Dirk Mehrens

**Affiliations:** 1 Department of Nuclear Medicine, LMU University Hospital, LMU Munich, Munich, Germany; 2 Department of Radiology, LMU University Hospital, LMU Munich, Munich, Germany; 3 Ahmanson Translational Theranostics Division, Department of Molecular and Medical Pharmacology, David Geffen School of Medicine, UCLA, California, Los Angeles USA; 4 Department of Radiology and Nuclear Medicine, Cancer Center Amsterdam, Amsterdam University Medical Center, Vrije Universiteit Amsterdam, Amsterdam, Netherlands; 5 Division of Nuclear Medicine, University Hospital Leuven and KU Leuven, Leuven, Belgium; 6 Nuclear Medicine Department, Humanitas Clinical and Research Hospital, Rozzano, Milan, Italy; 7 Department of Radiation Oncology, LMU University Hospital, LMU Munich, Munich, Germany; 8 Department of Urology, LMU University Hospital, LMU Munich, Munich, Germany

**Keywords:** PSMA PET/CT, Prostate cancer, Cost-effectiveness analysis, Primary staging, ProPSMA trial

## Abstract

**Purpose:**

The proPSMA trial at ten Australian centers demonstrated increased sensitivity and specificity for PSMA PET/CT compared to conventional imaging regarding metastatic status in primary high-risk prostate cancer patients. A cost-effectiveness analysis showed benefits of PSMA PET/CT over conventional imaging for the Australian setting. However, comparable data for other countries are lacking. Therefore, we aimed to verify the cost-effectiveness of PSMA PET/CT in several European countries as well as the USA.

**Methods:**

Clinical data on diagnostic accuracy were derived from the proPSMA trial. Costs for PSMA PET/CT and conventional imaging were taken from reimbursements of national health systems and individual billing information of selected centers in Belgium, Germany, Italy, the Netherlands, and the USA. For comparability, scan duration and the decision tree of the analysis were adopted from the Australian cost-effectiveness study.

**Results:**

In contrast to the Australian setting, PSMA PET/CT was primarily associated with increased costs in the studied centers in Europe and the USA. Mainly, the scan duration had an impact on the cost-effectiveness. However, costs for an accurate diagnosis using PSMA PET/CT seemed reasonably low compared to the potential consequential costs of an inaccurate diagnosis.

**Conclusion:**

We assume that the use of PSMA PET/CT is appropriate from a health economic perspective, but this will need to be verified by a prospective evaluation of patients at initial diagnosis.

## Main Report

**Fig. 1:**
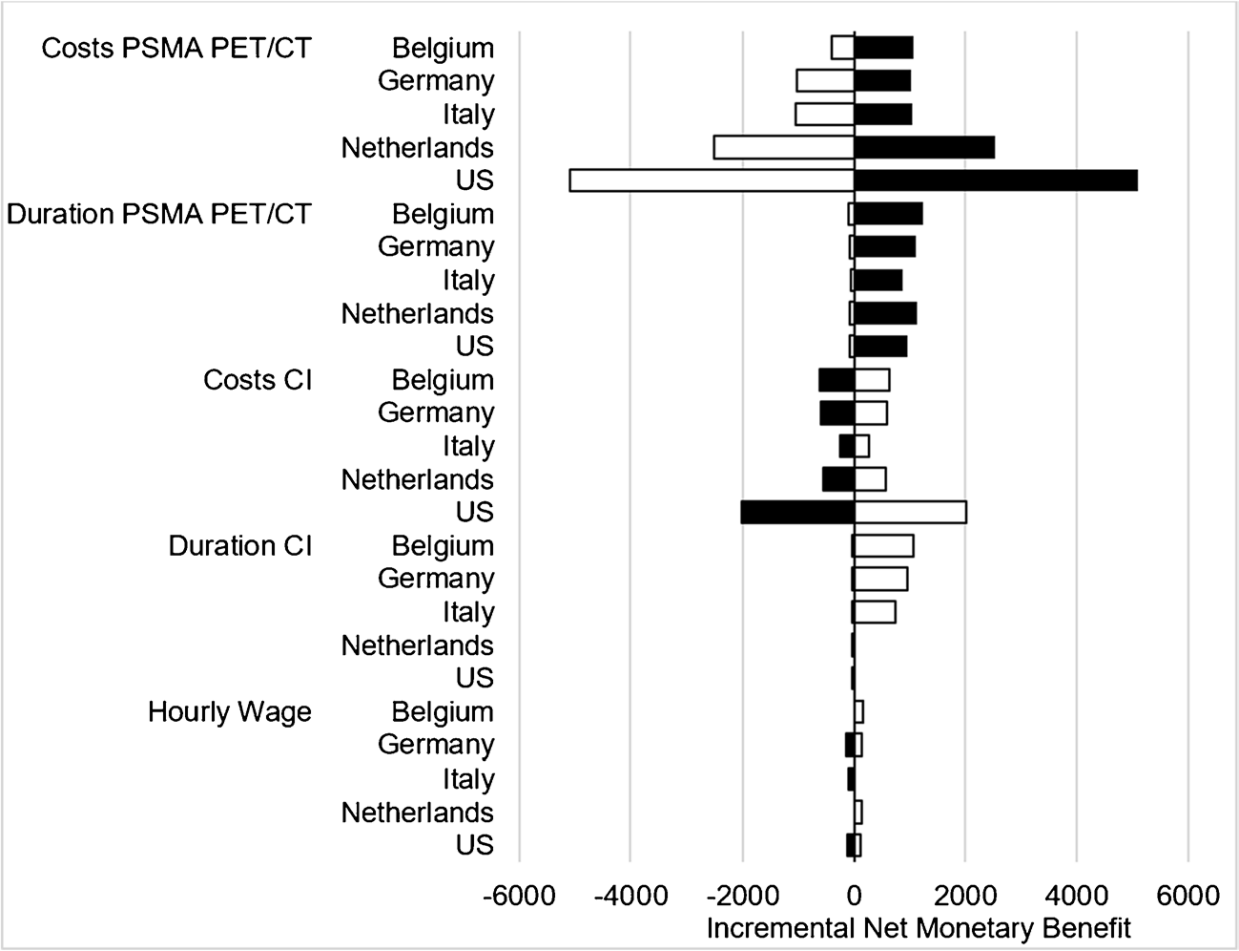
Tornado diagram for the deterministic sensitivity analysis. For comparison, impact of variables on cost-effectiveness of PSMA PET/CT were depicted as change in percentage from their respective base case results. Costs for PSMA PET/CT as well as duration for PSMA PET/CT demonstrated the strongest impact on the cost-effectiveness for PSMA PET/CT, followed by costs for CI as well as time needed for CI. Hourly wages only had a minor impact. Black bars indicate changes based on the upper bound of a parameter variation, white bars indicate the lower bound of the respective parameter (in EUR). Abbreviations: CI, conventional imaging; PSMA, Prostate-specific Membrane Antigen; PET/CT, Positron Emission Tomography/Computed Tomography.

Prostate-specific membrane antigen (PSMA) positron emission tomography (PET)/computed tomography (CT) has been adopted worldwide as an imaging modality for prostate cancer (PCa) and has shown a promising role for the initial staging [1, 2]. The proPSMA trial at ten Australian centers demonstrated increased sensitivity and specificity for PSMA PET/CT compared to conventional imaging, with respect to metastatic status in high-risk PCa patients [3]. A cost-effectiveness analysis showed benefits of PSMA PET/CT over conventional imaging for the Australian setting [4]. However, comparable data for other countries are lacking. Therefore, we aimed to verify the cost-effectiveness of PSMA PET/CT in several European countries as well as the USA. A decision tree was constructed using TreeAge Pro 2022 (Williamstown, USA), adopted from de Feria Cardet et al. [4]. Sensitivity and specificity for imaging modalities in patients with distant and nodal metastases were derived from the proPSMA trial [3]. Costs for PSMA PET/CT and conventional imaging (i.e. abdomen and pelvis CT with intravenous contrast, and whole body planar bone scan with single photon emission tomography of the chest to pelvis) were collected from national reimbursement rates and as information from single healthcare centers in Belgium, Germany, Italy, the Netherlands and the USA (see Table 1). In Belgium, Germany, Italy, and the USA reimbursement rates were available, for the Netherlands the single institution’s actual costs were used ([^18^F]-DCFPyL PSMA). For comparability, scan duration was also adopted from de Feria Cardet et al. [4]. Information on average hourly wages for patients were gathered from Eurostat (https://ec.europa.eu/eurostat/databrowser/view/lc_lci_lev, last accessed on January 08^th^, 2023) and the U.S. bureau of labor statistics (https://www.bls.gov/news.release/empsit.t19.htm, last accessed on January 08^th^, 2023). All costs were converted to 2022 national currencies (i.e., Euro and USD). Base case analysis and additional deterministic sensitivity analysis were conducted to account for uncertainties and to determine the impact of input parameters. In the selected centers in Europe and the USA, costs for PSMA PET/CT exceeded costs for conventional imaging, ranging from only minor differences in the Belgian center (incremental costs of 45 € per patient) to moderate differences in the German and the Italian centers (467 € and 834 €) and distinct differences in the Dutch and US-American centers (2,100 € and $ 3,277). Costs per additional diagnosis for PSMA PET/CT ranged from $14,714 (USA) to cost savings of 568 € (Belgium) (see Table 1 for results). The deterministic sensitivity analysis revealed that the costs of PSMA PET/CT as well as the time needed for PSMA PET/CT had the strongest impact on the results, followed by the costs and duration of conventional imaging. Hourly wages only had a minor effect on the results (see Figure 1). This is the first study to analyse cost-effectiveness of PSMA PET/CT compared to conventional imaging based on results of the proPSMA trial for European and the US-American settings. Although PSMA PET/CT was primarily associated with increased costs in the studied centers in Europe and the USA, costs for an accurate diagnosis using PSMA PET/CT seemed reasonably low compared to the potential consequential costs of an inaccurate diagnosis. For instance, in a patient with oligofocal osseous metastases, the early detection through PSMA PET/CT of otherwise imaging-occult additional nodal and visceral metastases could prevent unnecessary local treatments or costly radium-223 therapy and guide to an earlier start of appropriate systemic treatment [2, 5]. It should be noted that even within a single country, billing can widely vary from center to center, and the data presented can therefore only provide an exemplary insight. In Germany, for instance, PSMA PET/CT is not generally reimbursed via the health insurances system but multiple billing routes with potentially significant discrepancies in the claimed costs of a PSMA PET/CT scan exist [6]. A transparent overview of the range of cost amounts is not available. However, a uniform benchmark for some outpatient cases as well as fixed rates in the inpatient sector exist and are known to be comparable between institutions in Germany, therefore serving as a meaningful *pars pro toto* in the context of our analysis. Of note, reimbursement and average payment rates were available for the Belgian, the Italian, the German, and the US-American centers; the Dutch center provided a composite of production costs, material costs, personnel costs, and camera costs. Many other national circumstances and peculiarities make it difficult to draw a general comparison. For instance in Belgium, whose center was the only one to show cost savings with PSMA PET/CT, there is a different reimbursement for a recognized indication and for an orphan indication. The primary staging of PCa has just been accepted as a recognized indication by the scientific working group, but the according implementation into the reimbursement system still has to be realized. As an exemplary minor limitation, in some countries the reimbursement for contrast agent is dependent on the volume applied. In order not to unnecessarily increase the complexity of the model, standard volumes were used in the analysis in view of the rather small cost differences related to different volumes of contrast agent. Of note, the performed analysis is not a classical cost-effectiveness analysis trying to take into account all subsequent decision points and downstream costs based on outcomes, as there is not yet the level of evidence for modelling long-term effects. Therefore, in analogy to the Australian cost-effectiveness study, the cost per accurate diagnosis was chosen as the endpoint of our analysis [4, 7]. Also, a comparison across different health systems is not the scope of the study, but the results are bundled here rather within an editorial rationale to broadly inform the reader. In sum, PSMA PET/CT was primarily associated with increased costs in the studied centers in Europe and the USA, in contrast to the Australian setting [4]. However, we assume that the use of PSMA PET/CT is appropriate from a health economic perspective as the costs for an accurate diagnosis using PSMA PET/CT seemed reasonably low compared to the potential consequential costs of an inaccurate diagnosis. This will need to be verified by a prospective evaluation of patients at initial diagnosis.

**Table 1:** This table includes the input parameters used for the analysis as well as the results of the study including costs of imaging, incremental costs, and costs per additional diagnosis

	PSMA PET/CT	Conventional Imaging
Distant Metastases		
Sensitivity, % (95% Confidence Interval)	92 (81-100)	54 (34-74)
Specificity, % (95% Confidence Interval)	99 (98-100)	93 (88-97)
Nodal Metastases		
Sensitivity, % (95% Confidence Interval)	83 (70-95)	23 (10-35)
Specificity, % (95% Confidence Interval)	99 (97-100)	96 (93-100)
Costs of Imaging (± 20% for the sensitivity analysis)		
Belgium	719 € (575-863)	674 € (539-809)
Germany	1,102 € (882-1,322)	635 € (508-762)
Italy	1,116 € (893-1,339)	282 € (226-338)
Netherlands	2,700 € (2,160-3,240)	600 € (480-720)
USA	$ 5,438 (4,350-6,526)	$ 2,161 (1,729-2,593)
Incremental Costs		
Belgium	45 €	-
Germany	467 €	-
Italy	834 €	-
Netherlands	2,100 €	-
USA	$ 3,277	-
Scan Duration (Hours)	1.51 (0.07-5.75)	5.52 (1-7.83)
Hourly Wages		
Belgium	41,6 € (33-50)	
Germany	37,2 € (30-44.6)	
Italy	29,3 € (23.4-35)	
Netherlands	38,3 € (30.6-46)	
USA	$ 32,5 (26-39)	
Costs Per Additional Diagnosis (nodal or distant metastasis)		
Belgium	-568 €	-
Germany	1,488 €	-
Italy	3,351 €	-
Netherlands	9,102 €	-
USA	$ 14,714	-

Conventional Imaging = abdomen and pelvis CT with intravenous contrast, CT and whole body planar bone scan with single photon emission tomography of the chest to pelvis. PSMA PET/CT = reimbursement and average payment rates were available for the Belgian, the Italian, the German, and the US-American centers; the Dutch center provided a composite of production costs, material costs, personnel costs, and camera costs.

## Data Availability

The datasets generated and/or analyzed during the current study are mainly given in the manuscript. Further data may be available from the corresponding author on reasonable request.
